# Supplementary material for: The Molecular Profile of Soil Microbial Communities Inhabiting a Cambrian Host Rock
Source: Microorganisms. 2024 Mar 2;12(3):513. doi: 10.3390/microorganisms12030513 (PMC10975187; doi:10.3390/microorganisms12030513)
Supplement: Supplementary file 1 [file microorganisms-12-00513-s001.zip › microorganisms-2810772-supplementary.pdf]

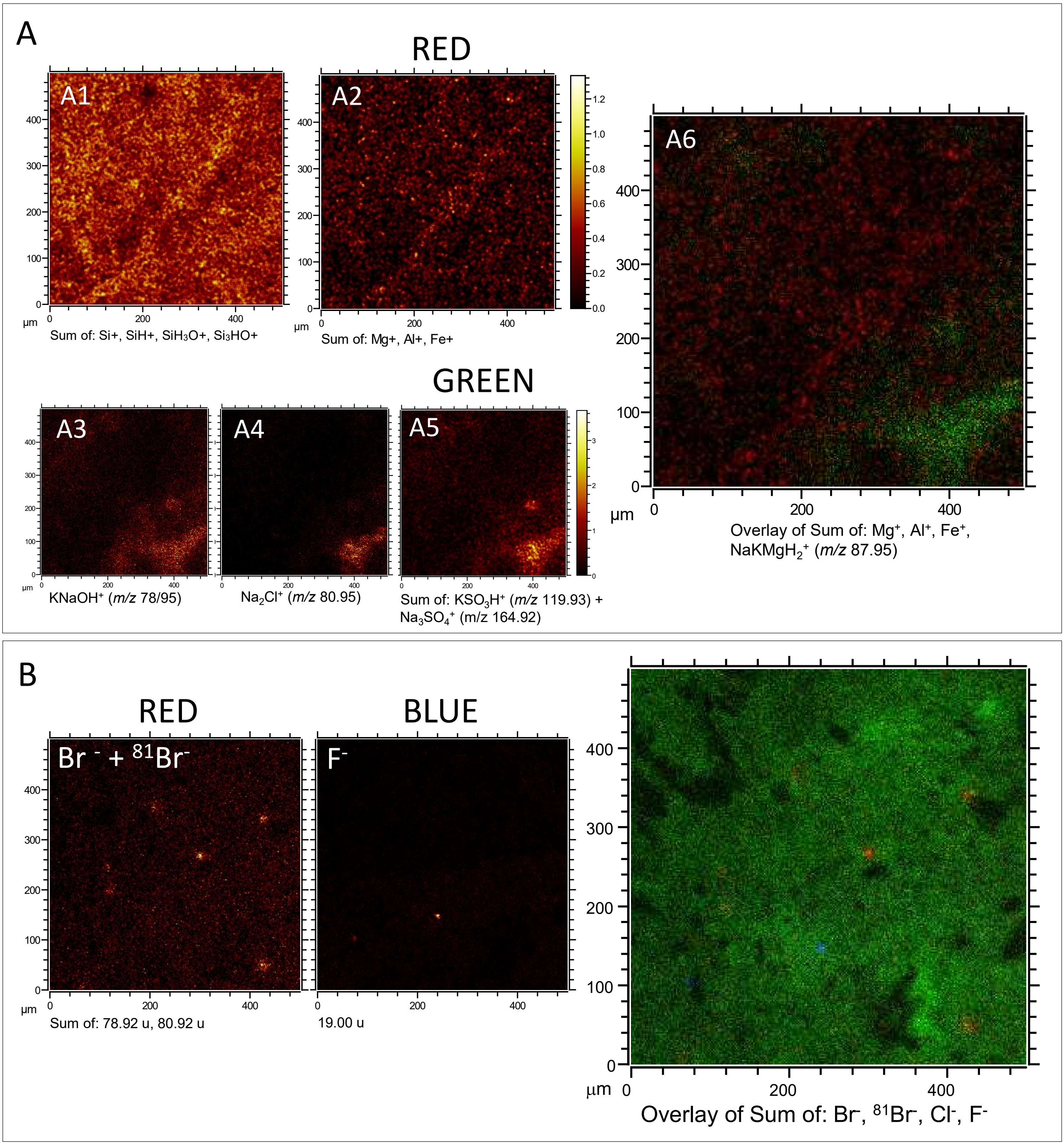
A
RED
A1
Sum of: Si+, SiH+, SiH3O+, Si3HO+
A2
Sum of: Mg+, Al+, Fe+
A6
Overlay of Sum of: Mg+, Al+, Fe+, NaKMgH2+ (m/z 87.95)
GREEN
A3
KNaOH+ (m/z 78/95)
A4
Na2Cl+ (m/z 80.95)
A5
Sum of: KSO3H+ (m/z 119.93) + Na3SO4+ (m/z 164.92)
µm
B
RED
Br - + 81Br-
Sum of: 78.92 u, 80.92 u
BLUE
F-
19.00 u
Overlay of Sum of: Br-, 81Br-, Cl-, F-

Figure S1.

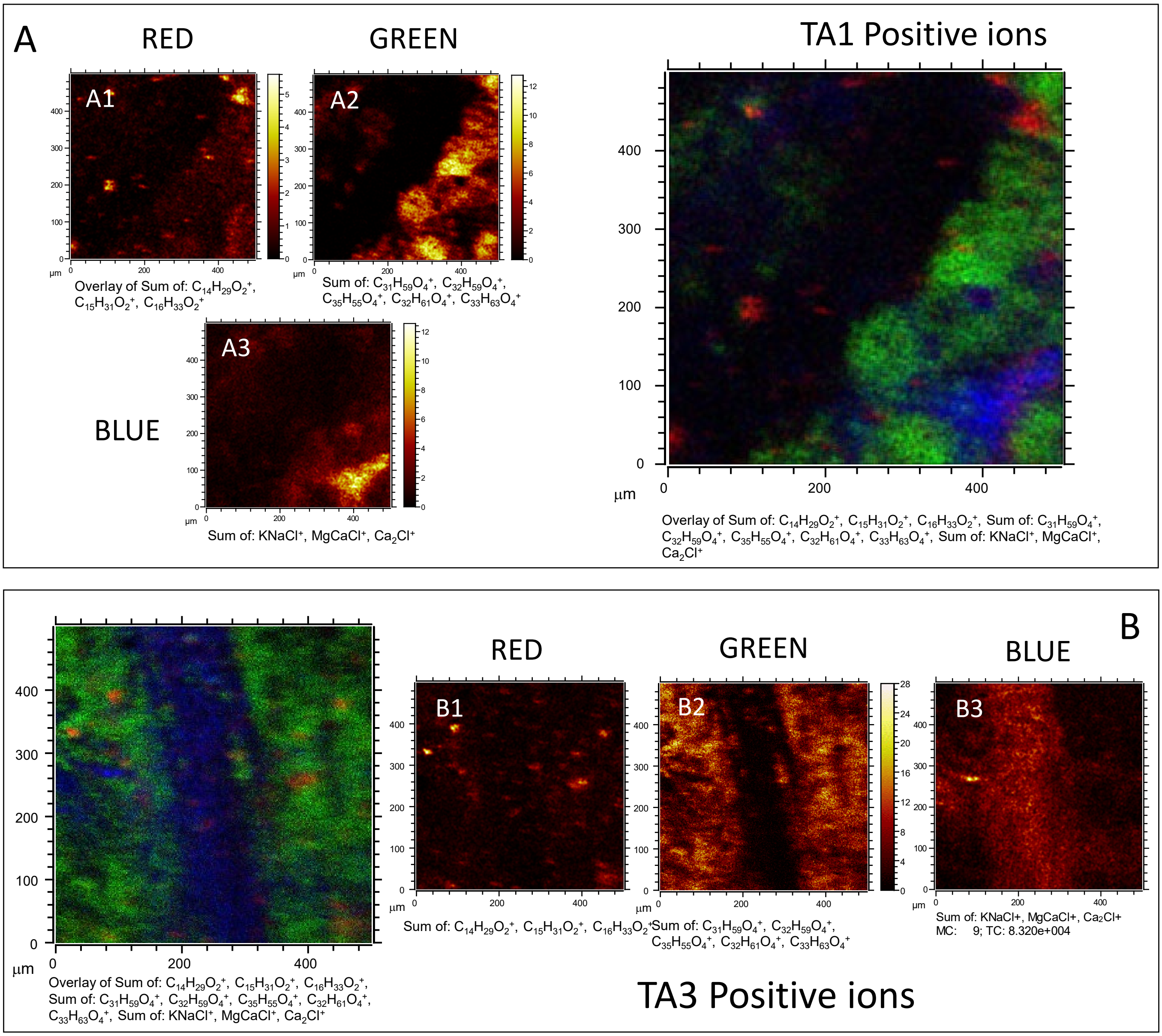
A
RED
GREEN
BLUE
A1
A2
A3
TA1 Positive ions
Overlay of Sum of: $C_{14}H_{29}O_2^+$, $C_{15}H_{31}O_2^+$, $C_{16}H_{33}O_2^+$
Sum of: $C_{31}H_{59}O_4^+$, $C_{32}H_{59}O_4^+$, $C_{35}H_{55}O_4^+$, $C_{32}H_{61}O_4^+$, $C_{33}H_{63}O_4^+$
Sum of: $KNaCl^+$, $MgCaCl^+$, Ca_2Cl^+
Overlay of Sum of: $C_{14}H_{29}O_2^+$, $C_{15}H_{31}O_2^+$, $C_{16}H_{33}O_2^+$, Sum of: $C_{31}H_{59}O_4^+$, $C_{32}H_{59}O_4^+$, $C_{35}H_{55}O_4^+$, $C_{32}H_{61}O_4^+$, $C_{33}H_{63}O_4^+$, Sum of: $KNaCl^+$, $MgCaCl^+$, Ca_2Cl^+
µm
B
RED
GREEN
BLUE
B1
B2
B3
Overlay of Sum of: $C_{14}H_{29}O_2^+$, $C_{15}H_{31}O_2^+$, $C_{16}H_{33}O_2^+$, Sum of: $C_{31}H_{59}O_4^+$, $C_{32}H_{59}O_4^+$, $C_{35}H_{55}O_4^+$, $C_{32}H_{61}O_4^+$, $C_{33}H_{63}O_4^+$, Sum of: $KNaCl^+$, $MgCaCl^+$, Ca_2Cl^+
Sum of: $C_{14}H_{29}O_2^+$, $C_{15}H_{31}O_2^+$, $C_{16}H_{33}O_2^+$
Sum of: $C_{31}H_{59}O_4^+$, $C_{32}H_{59}O_4^+$, $C_{35}H_{55}O_4^+$, $C_{32}H_{61}O_4^+$, $C_{33}H_{63}O_4^+$
Sum of: KNaCl+, MgCaCl+, Ca2Cl+
MC: 9; TC: 8.320e+004
TA3 Positive ions

Figure S2.

Table S1. *n*-Fatty acid list extracted from sample 95E5/2 using GC-MS

Acid fraction		
**Compound (as methyl esters)**	**Molecular formula as methyl esters**	**Fatty acid formula**
Butanoic acid, 3-hydroxy-, methyl ester	$C_5H_{10}O_3$	$C_4H_8O_3$
Hexanoic acid, methyl ester	$C_7H_{14}O_2$	$C_6H_{12}O_2$
Octanoic acid, methyl ester	$C_9H_{18}O_2$	$C_8H_{16}O_2$
Nonanoic acid, methyl ester	$C_{10}H_{20}O_2$	$C_9H_{18}O_2$
Hexanedioic acid, dimethyl ester	$C_8H_{14}O_4$	$C_6H_{10}O_4$
Decanoic acid, methyl ester	$C_{11}H_{22}O_2$	$C_{10}H_{20}O_2$
Methyl 8-oxooctanoate, methyl ester	$C_9H_{16}O_3$	$C_8H_{16}O_3$
Heptanedioic acid, dimethyl ester	$C_9H_{16}O_4$	$C_7H_{12}O_4$
Methyl 3,4-di-O-methyl-β-L-arabinopyranoside	$C_9H_{16}O_5$	$C_6H_{12}O_5$
Undecanoic acid, methyl ester	$C_{12}H_{24}O_2$	$C_{11}H_{22}O_2$
Nonanoic acid, 9-oxo-, methyl ester	$C_{10}H_{18}O_3$	$C_9H_{16}O_3$
Octanedioic acid, dimethyl ester	$C_{10}H_{18}O_4$	$C_8H_{14}O_4$
Nonanedioic acid, dimethyl ester	$C_{11}H_{20}O_4$	$C_9H_{16}O_4$
Dodecanoic acid, 4-methyl-, methyl ester (*i* C_{12})	$C_{14}H_{28}O_2$	$C_{13}H_{26}O_2$
Tridecanoic acid, methyl ester	$C_{14}H_{28}O_2$	$C_{13}H_{26}O_2$
Dodecanoic acid, 10-methyl-, methyl ester (*a* C_{12})	$C_{14}H_{28}O_2$	$C_{13}H_{26}O_2$
Decanedioic acid, dimethyl ester	$C_{12}H_{22}O_4$	$C_{10}H_{18}O_4$
Tridecanoic acid, 12-methyl-, methyl ester (*i* C_{13})	$C_{15}H_{30}O_2$	$C_{14}H_{28}O_2$
Tetradecanoic acid, methyl ester	$C_{15}H_{30}O_2$	$C_{14}H_{26}O_2$
Undecanedioic acid, dimethyl ester	$C_{13}H_{24}O_4$	$C_{11}H_{20}O_4$
Tetradecanoic acid, 12-methyl-, methyl ester (*i* C_{14})	$C_{16}H_{32}O_2$	$C_{15}H_{30}O_2$
Methyl 13-methyltetradecanoate, methyl ester (*i* C_{14})	$C_{16}H_{32}O_2$	$C_{15}H_{30}O_2$
Pentadecanoic acid, methyl ester	$C_{16}H_{32}O_2$	$C_{15}H_{30}O_2$
Methyl hexadec-9-enoate	$C_{17}H_{32}O_2$	$C_{16}H_{32}O_2$
Hexadecanoic acid, methyl ester	$C_{17}H_{34}O_2$	$C_{16}H_{32}O_2$
Hexadecanoic acid, 15-methyl-, methyl ester (*a* C16)	$C_{18}H_{36}O_2$	$C_{17}H_{34}O_2$
Hexadecanoic acid, 14-methyl-, methyl ester (*i* C16)	$C_{18}H_{36}O_2$	$C_{17}H_{34}O_2$
Heptadecanoic acid, methyl ester	$C_{18}H_{36}O_2$	$C_{17}H_{34}O_2$
Octadecanoic acid, methyl ester	$C_{19}H_{38}O_2$	$C_{18}H_{36}O_2$
Octadecanoic acid, methyl ester	$C_{19}H_{38}O_2$	$C_{18}H_{36}O_2$
11-Octadecenoic acid, methyl ester	$C_{19}H_{36}O_2$	$C_{18}H_{34}O_2$
Nonadecanoic acid, methyl ester	$C_{20}H_{40}O_2$	$C_{20}H_{40}O_2$
Eicosanoic acid, methyl ester	$C_{21}H_{42}O_2$	$C_{20}H_{40}O_2$
Heneicosanoic acid, methyl ester	$C_{22}H_{44}O_2$	$C_{21}H_{42}O_2$
Docosanoic acid, methyl ester	$C_{23}H_{46}O_2$	$C_{22}H_{44}O_2$
Tricosanoic acid, methyl ester	$C_{24}H_{48}O_2$	$C_{23}H_{46}O_2$
Tetracosanoic acid, methyl ester	$C_{25}H_{50}O_2$	$C_{24}H_{48}O_2$
Pentacosanoic acid, methyl ester	$C_{26}H_{52}O_2$	$C_{25}H_{50}O_2$
Hexacosanoic acid, methyl ester	$C_{27}H_{54}O_2$	$C_{26}H_{52}O_2$
Tricosanoic acid, 10,14,18,22-tetramethyl-, methyl ester	$C_{28}H_{56}O_2$	$C_{27}H_{54}O_2$
Octacosanoic acid, methyl ester	$C_{29}H_{58}O_2$	$C_{28}H_{56}O_2$

Table S2. Branched, unsaturated and dicarboxylic acids from sample 95E5/2 using GC-MS.

Iso/anteiso fatty acid	Molecular formula as methyl esters	Alkanoic acid formula	Reference name
Methyl 11-methyl-dodecanoate	C14H28O2	C13H26O2	11-isoC12
Dodecanoic acid, 10-methyl-, methyl ester	C14H28O2	C13H26O2	12-anteisoC12
Tridecanoic acid, 12-methyl-, methyl ester	C15H30O2	C14H28O2	12-isoC13
Methyl 13-methyltetradecanoate	C17H34O2	C16H32O2	13-isoC15
Tetradecanoic acid, 12-methyl-, methyl ester	C17H34O2	C16H32O2	12-anteisoC15
Hexadecanoic acid, 15-methyl-, methyl ester	C18H36O2	C17H34O2	15-isoC17
Hexadecanoic acid, 14-methyl-, methyl ester	C18H36O2	C17H34O2	14-anteisoC17
Methyl 20-methyl-heneicosanoate	C23H46O2	C22H44O2	20-isoC21
Methyl 21-methyl-heneicosanoate	C23H46O2	C22H44O2	21-anteisoC21
Methyl 21-methyldocosanoate	C24H48O2	C23H46O2	21-isoC22
Methyl 20-methyl-docosanoate	C24H48O2	C23H46O2	20-anteisoC22
Methyl 21-methyl-tetracosanoate	C26H52O2	C25H50O2	21-isoC24
Methyl 22-methyl-tetracosanoate	C26H52O2	C25H50O2	22-anteisoC24
Methyl indet methyl-pentacosanoate	C27H54O2	C26H52O2	IsoC25
Methyl 20-methyl-hexacosanoate	C28H56O2	C27H54O2	20-isoC27
Methyl 21-methyl-hexacosanoate	C28H56O2	C27H54O2	21-anteisoC27

Methyl branched fatty acid	Molecular formula as methyl esters	Alkanoic acid formula	Reference name
Methyl 8-oxooctanoate			
Undecanoic acid, 10-methyl-, methyl ester	C13H26O2	C12H24O2	10Me-C11
Dodecanoic acid, 4-methyl-, methyl ester	C14H28O2	C13H26O2	4Me-C12
Methyl Z-11-tetradecenoate	C16H30O2	C14H26O2	11Me-C14:1
Pentadecanoic acid, 14-methyl-, methyl ester	C17H34O2	C16H32O2	14Me-C15
Pentadecanoic acid, 13-methyl-, methyl ester	C17H34O2	C16H32O2	13Me-C15
Methyl 10-methyl-hexadecanoate	C18H36O2	C17H34O2	10Me-C16

Unsaturated n-FAs	Molecular formula as methyl esters	Alkanoic acid formula	Reference name
9,12-Octadecadienoic acid (Z,Z)-, methyl ester	C19H34O2	C18H32O2	C18:2w9,12
9-Octadecenoic acid (Z)-, methyl ester	C19H36O2	C18H34O2	C18:1w9
11-Octadecenoic acid, methyl ester	C19H36O2	C18H34O2	C18:1w11

Dicarboxylic fatty acids	Molecular formula as methyl esters	Alkanoic acid formula	Reference name
Hexanedioic acid, dimethyl ester	C8H14O4	C6H10O4	di-C16
Octanedioic acid, dimethyl ester	C10H18O4	C8H14O4	di-C18
Nonanedioic acid, dimethyl ester	C11H20O4	C9H16O4	di-C19
Docosanedioic acid, dimethyl ester	C24H46O4	C22H42O4	di-C22

Table S3. Polar fraction extracted from sample 95E5/2 using GC-MS.

Polar fraction		
**Compound**	**TMS molecular formula**	**Molecular formula**
2-Ethylhexanol, TMS derivative	$C_{11}H_{26}OSi$	$C_8H_{18}O$
3-Octen-2-ol, (E)-, TMS derivative	$C_{11}H_{24}OSi$	$C_8H_{16}O$
2-Octene, 2-(trimethylsilyloxy)-	$C_{11}H_{24}OSi$	$C_8H_{16}O$
Diethylene glycol, 2TMS derivative	$C_{10}H_{26}O_3Si_2$	$C_4H_{10}O_3$
Glycerol, 3TMS derivative	$C_{12}H_{32}O_3Si_3$	$C_3H_8O_3$
1,12-Dodecanediol, 2TMS derivative	$C_{18}H_{42}O_2Si_2$	$C_{12}H_{26}O_2$
Benzene, 2,4-diisocyanato-1-methyl-	$C_9H_6N_2O_2$	$C_9H_6N_2O_2$
phenoxyethanol, TMS derivative	$C_{11}H_{18}O_2Si$	$C_8H_{10}O_2$
1-Undecanol, TBDMS derivative	$C_{17}H_{38}OSi$	$C_{11}H_{24}O$
Triethylene glycol, 2TMS derivative	$C_{12}H_{30}O_4Si_2$	$C_6H_{14}O_4$
Tripropylene glycol monomethyl ether, TMS derivative	$C_{13}H_{30}O_4Si$	$C_7H_{16}O_3$
1-Dodecanol, TMS derivative	$C_{15}H_{34}OSi$	$C_{12}H_{26}O$
1-Tridecanol, TMS derivative	$C_{16}H_{36}OSi$	$C_{13}H_{28}O$
Pimelic acid, 2TMS derivative	$C_{13}H_{28}O_4Si_2$	$C_7H_{12}O_4$
2-Phenylisopropanol, TMS derivative	$C_{12}H_{20}OSi$	$C_9H_{12}O$
1-Tetradecanol, TMS derivative	$C_{17}H_{38}OSi$	$C_{14}H_{30}O$
1-Pentadecanol, TMS derivative	$C_{18}H_{40}OSi$	$C_{15}H_{32}O$
1-Hexadecanol, TMS derivative	$C_{19}H_{42}OSi$	$C_{16}H_{34}O$
1-Heptadecanol, TMS derivative	$C_{20}H_{44}OSi$	$C_{17}H_{36}O$
1-Octadecanol, TMS derivative	$C_{21}H_{46}OSi$	$C_{18}H_{38}O$
1-Nonadecanol, TMS derivative	$C_{22}H_{48}OSi$	$C_{19}H_{40}O$
18-Methyl-nonadecanol, trimethylsilyl ether	$C_{23}H_{50}OSi$	$C_{20}H_{42}O$
1-Heneicosanol, TMS derivative	$C_{24}H_{52}OSi$	$C_{21}H_{44}O$
1-Monopalmitoylglycerol trimethylsilyl ether	$C_{25}H_{54}O_4Si_2$	$C_{19}H_{38}O_4$
1-Tricosanol, TMS derivative	$C_{26}H_{56}OSi$	$C_{23}H_{48}O$
1-Tetracosanol, TMS derivative	$C_{27}H_{58}OSi$	$C_{24}H_{50}O$
Glycerol monostearate, 2TMS derivative	$C_{27}H_{58}O_4Si_2$	$C_{21}H_{42}O_4$
1-Hexacosanol, TMS derivative	C_{29}H62OSi	$C_{26}H_{54}O$
1-Octacosanol, TMS derivative	$C_{31}H_{66}OSi$	$C_{28}H_{56}O$
1-Monooleoylglycerol, 2TMS derivative	$C_{27}H_{56}O_4Si_2$	$C_{21}H_{40}O_4$
Benzenepropanoic acid, 3,5-bis(1,1-dimethylethyl)-4-trimethylsilyloxy-, octadecyl ester	$C_{38}H_{70}O_3Si$	$C_{18}H_{28}O_3$

Sterols		
**Compound**	**TMS derivative formula**	**Molecular formula**
Cholesta-4,6-dien-3-ol, (3β)- (cholestadienol=desmosterol)	$C_{30}H_{52}OSi$	$C_{27}H_{44}O$
Cholest-5-en-3-ol (cholesterol)	$C_{30}H_{54}OSi$	$C_{27}H_{46}O$
Ergosta-5,22-dien-3-ol, acetate, (3β,22E)- (ergostadienol)	$C_{31}H_{54}OSi$	$C_{28}H_{46}O$
Ergost-5-en-3 beta- ol, 24S (campesterol)	$C_{31}H_{56}OSi$	$C_{28}H_{48}O$
Stigmast-5-en-3-ol, (3β,24S)- (stigmastenol/b-sitosterol)	$C_{32}H_{58}OSi$	$C_{29}H_{50}O$
Stigmastanol, (3β,5β,24S) (stigmastanol)	$C_{32}H_{60}OSi$	$C_{29}H_{52}O$

Table S4. Organic ratios utilized for unraveling the origins of n-alkanes and n-fatty acids from the GC/MS data.

Name	Equation	Result	Interpetation
Average Carbon Length (ACL)	$ACL = \frac{\sum_{n=11}^{33} n \cdot C_n}{\sum_{n=11}^{33} C_n}$	$n - alkanoic\ acids$ $ACL = 18$	derived from microbial sources [1]
Carbon Preference Index (CPI)	$CPI = \frac{1}{2}\left(\frac{C_{13}+C_{15}+C_{17}+C_{19}+C_{21}+C_{23}+C_{25}+C_{27}+C_{29}+C_{31}+C_{33}}{C_{12}+C_{14}+C_{16}+C_{18}+C_{20}+C_{22}+C_{24}+C_{26}+C_{28}+C_{30}+C_{32}}\right) + \frac{1}{2}\left(\frac{C_{13}+C_{15}+C_{17}+C_{19}+C_{21}+C_{23}+C_{25}+C_{27}+C_{29}+C_{31}+C_{33}}{C_{14}+C_{16}+C_{18}+C_{20}+C_{22}+C_{24}+C_{26}+C_{28}+C_{30}+C_{32}}\right)$	$n - alkanoic\ acids$ $CPI = 3.73$	extant biomass [2, 3]
Prokaryotic over eukaryotic sources (LMW/HMW)	$where\ LMW \leq nC_{20}\ and\ HMW \geq nC_{21}$ $\frac{LMW}{HMW} = \frac{\sum_{n=12}^{20} C_n}{\sum_{m=21}^{34} C_m}$	n-alkanoic acids $LMW/HMW = 5.46$	predominance of prokaryotic origin [4, 5]

Table S5. $[M + H - H2O]^+$ and $[M - H]^-$ fragments of fatty acids indentified through ToF-SIMS in sample 95E5/3

FA	m/z	$[M + H - H_2O]^+$	error (ppm)	Intensity (cps)	m/z	$[M - H]^-$	error (ppm)	FA	Intensity (cps)
C4:1	69.03	C4H5O+	4.87	2184	85.03	C4H5O2-	9.54	C4:1	-
C4:0	71.05	C4H7O+	8.64	2029	87.05	C4H7O2-	12.75	C4:0	979
C5:1	83.05	C5H7O+	3.63	1872	99.05	C5H7O2-	26.65	C5:1	1506
C5:0	85.06	C5H9O+	-1.63	1005	101.06	C5H9O2-	-18.87	C5:0	-
C6:2	95.05	C6H7O+	21.58	984	111.05	C6H7O2-	11.46	C6:2	-
C6:1	97.07	C6H9O+	42.49	1280	113.06	C6H9O2-	15.72	C6:1	-
C6:0	99.08	C6H11O+	-1.61	598	115.08	C6H11O2-	4.36	C6:0	475
C7:1	111.09	C7H11O+	47.14	601	127.08	C7H11O2-	0.00	C7:1	1473
C7:0	113.09	C7H13O+	-10.18	218	129.09	C7H13O2-	12.20	C7:0	348
C8:1	123.10	C8H13O+	14.83	242	141.09	C8H13O2-	9.10	C8:1	1101
C8:0	125.10	C8H15O+	30.23	242	143.11	C8H15O2-	-8.71	C8:0	377
C9:2	137.10	C9H13O+	2.76	180	153.09	C9H13O2-	-5.51	C9:2	221
C9:1	139.11	C9H15O+	-7.76	91	155.11	C9H15O2-	1.79	C9:1	874
C9:0	141.13	C9H17O+	3.31	70	157.12	C9H17O2-	-25.64	C9:0	311
C10:2	151.11	C10H15O+	13.36	84	167.10	C10H15O2-	-15.08	C10:2	186
C10:1	153.13	C10H17O+	-0.53	57	169.12	C10H17O2-	-2.81	C10:1	683
C10:0	155.14	C10H19O+	-6.88	78	171.14	C10H19O2-	-18.31	C10:0	428
C11:1	167.14	C11H19O+	20.47	48	183.14	C11H19O2-	-0.46	C11:1	604
C11:0	169.16	C11H21O+	27.98	32	185.15	C11H21O2-	-24.30	C11:0	180
C12:1	-	-	-	-	197.15	C12H21O2-	-14.90	C12:1	460
C12:0	183.18	C12H23O+	15.03	103	199.17	C12H23O2-	-8.79	C12:0	785
C13:2	-	-	-	-	209.19	C13H21O2-	20.11	C13:2	134
C13:1	195.18	C13H23O+	22.32	29	211.17	C13H23O2-	0.10	C13:1	393
C13:0	197.19	C13H25O+	12.32	43	213.18	C13H25O2-	-20.65	C13:0	407
C14:1	209.19	C14H25O+	20.11	78	225.19	C14H25O2-	0.34	C14:1	829
C14:0	211.22	C14H27O+	45.24	2015	227.20	C14H27O2-	-4.53	C14:0	5006
C15:1	223.21	C15H27O+	12.17	72	239.20	C15H27O2-	13.78	C15:1	581
C15:0	225.24	C15H29O+	20.55	132	241.22	C15H29O2-	-8.87	C15:0	3822
C16:2	235.22	C16H27O+	45.62	60	251.21	C16H27O2-	18.74	C16:2	301
C16:1	237.22	C16H29O+	37.83	148	253.22	C16H29O2-	-4.40	C16:1	2130
C16:0	239.25	C16H31O+	40.77	398	255.23	C16H31O2-	-7.12	C16:0	12537
C17:1	251.24	C17H31O+	29.64	40	267.23	C17H31O2-	-6.73	C17:1	469
C17:0	253.25	C17H33O+	32.45	31	269.24	C17H33O2-	-24.72	C17:0	1330
C18:4	-	-	-	-	275.20	C18H27O2-	1.73	C18:4	25
C18:3	-	-	-	-	277.21	C18H29O2-	-14.76	C18:3	53
C18:2	263.24	C18H31O+	23.04	31	279.23	C18H31O2-	-16.52	C18:2	197
C18:1	265.25	C18H33O+	38.07	57	281.24	C18H33O2-	0.51	C18:1	774
C18:0	267.27	C18H35O+	61.60	74	283.26	C18H35O2-	-52.44	C18:0	1974
C19:1	279.26	C19H35O+	-20.15	41	295.26	C19H35O2-	4.02	C19:1	90
C19:0	-	-	-	-	297.26	C19H37O2-	-33.58	C19:0	168
C20:2	-	-	-	-	307.27	C20H35O2-	-19.65	C20:2	53
C20:1	-	-	-	-	309.27	C20H37O2-	-31.43	C20:1	128
C20:0	-	-	-	-	311.29	C20H39O2-	-22.58	C20:0	198
C21:0	-	-	-	-	325.30	C21H41O2-	-32.07	C21:0	63
C22:0	-	-	-	-	339.32	C22H43O2-	-25.71	C22:0	190
C23:0	-	-	-	-	353.33	C23H45O2-	-22.39	C23:0	112
C24:1	-	-	-	-	365.33	C24H45O2-	-26.53	C24:1	78
C24:0	-	-	-	-	367.35	C24H47O2-	-21.81	C24:0	408
C25:1	-	-	-	-	379.35	C25H47O2-	-24.52	C25:1	47
C25:0	-	-	-	-	381.36	C25H49O2-	-40.99	C25:0	183
C26:1	-	-	-	-	393.37	C26H49O2-	-49.63	C26:1	51
C26:0	-	-	-	-	395.38	C26H51O2-	-75.75	C26:0	194
C27:0	-	-	-	-	409.39	C27H53O2-	37.80	C27:0	47
C28:0	-	-	-	-	423.40	C28H55O2-	-5.50	C28:0	41
C29:0	-	-	-	-	437.40	C29H57O2-	-75.75	C29:0	11
C30:1	-	-	-	-	449.45	C30H57O2-	37.80	C30:1	53
C32:1	-	-	-	-	477.47	C32H61O2-	-5.50	C32:1	34

Table S6. Fragments of glycerides and wax esters identified by Tof-SIMS in sample 95E5/3

DG major cations			
m/z	$[M + H - H_2O]^+$	error (ppm)	Intensity (cps)
409.34	C25H45O4+	23.85	59
411.37	C25H47O4+	51.58	40
421.33	C26H47O4+	5.22	30
423.35	C26H49O4+	17.05	46
425.38	C26H49O4+	37.62	45
435.35	C27H47O4+	3.21	22
437.37	C27H49O4+	7.43	51
439.39	C27H51O4+	25.03	91
453.41	C28H53O4+	41.09	31
465.40	C29H53O4+	17.21	64
467.42	C29H55O4+	14.78	105
479.41	C30H55O4+	0.65	55
481.44	C30H57O4+	36.22	62
493.43	C31H57O4+	15.92	101
495.45	C31H59O4+	21.02	307
509.46	C32H61O4+	11.35	316
521.46	C33H61O4+	14.48	327
523.48	C33H63O4+	17.07	1290
535.48	C34H63O4+	20.60	275
537.50	C34H65O4+	22.11	758
549.51	C35H65O4+	32.40	495
551.51	C35H67O4+	13.66	1729
563.51	C36H67O4+	15.24	145
565.53	C36H69O4+	16.64	354
577.53	C37H69O4+	25.34	206
579.54	C37H71O4+	17.78	418
591.55	C38H71O4+	29.89	63
593.57	C38H73O4+	28.42	74
605.56	C39H73O4+	13.34	79
607.58	C39H75O4+	14.81	156
619.59	C40H75O4+	38.11	24
621.60	C40H77O4+	58.91	35
635.62	C41H79O4+	28.66	61
647.61	C42H79O4+	13.11	28
649.62	C42H81O4+	13.02	58
663.62	C43H85O4+	-15.12	78
677.65	C44H87O4+	3.27	50
691.67	C45H87O4+	7.73	41
705.70	C46H89O4+	28.64	22
719.70	C47H91O4+	15.09	18
733.71	C48H93O4+	10.24	15
747.71	C49H95O4+	-19.84	11

Wax esters			
m/z	$[M + H - H_2O]^+$	error (ppm)	Intensity (cps)
229.22	$C_{14}H_{29}O_2^+$	14.16	89
243.22	$C_{15}H_{31}O_2^+$	-51.00	69
257.25	$C_{16}H_{33}O_2^+$	7.56	248

Correspondence between the most abundant diacylglycerides and their fragments obtained via ToF-SIMS					
Exact mass	Diglyceride formula	Diglyceride structure	m/z	Ion	error (ppm)
540.48	C33H64O5	DG(16:0/14:0)	211.21	C14H27O+	45.24
			239.25	C16H31O+	40.77
			241.22	C15H29O2+	-7.28
			243.24	C15H31O2+/C16H31O2-	41.03
			255.23	C16H31O2+/C16H31O2-	9.39
			285.25	C17H33O3+	54.11
			313.29	C19H37O3+	42.55
			225.23	C18H29O+	60.97
554.49	C34H66O5	DG(16:0/15:0)	225.23	C15H29O+	60.97
			239.25	C16H31O+	40.77
			255.23	C16H31O2+/C16H31O2-	9.39
			299.27	C18H35O3+	23.32
568.51	C35H68O5	DG(16:0/16:0)	239.25	C16H31O+	40.77
			255.23	C16H31O2+	9.39
			269.25	C17H33O2+	19.00
			283.27	C18H35O2+	39.29
			299.27	C18H35O3+	23.32
			313.29	C19H37O3+	42.55
594.52	C37H70O5	DG(18:1/16:0)	239.25	C16H31O+	40.77
			255.23	C16H31O2-	9.39
			265.26	C18H33O+	38.07
			281.24	C18H33O2-	-12.81
			299.27	C18H35O3+	23.32
596.54	C37H72O5	DG(18:0/16:0)	239.25	C16H31O+	40.77
			255.23	C16H31O2-	9.39
			341.30	C21H41O3+	5.74
			299.27	C18H35O3+	23.32
			297.28	C19H37O2+	14.04
			313.29	C19H37O3+	42.55
			267.28	C18H35O+	19.86
610.55	C38H74O5	DG(17:0/18:0)	253.25	C17H33O+	-6.48
			267.28	C18H35O+	28.10
			269.24	C17H33O2+	19.00
			283.26	C18H35O2+	39.29
			299.27	C18H35O3+	23.32
			313.29	C19H37O3+	40.80
			327.30	C20H39O3+	11.82
			341.30	C21H41O3+	5.74

Table S7. Sterol and hopanoid fragments of sample 95E5/3 collected in the Pedroche Fm through ToF-SIMS.

m/z	intensity (cps)	Tentative formula	error (ppm)	Compound	
149.13	205	C11H17+	0.23	B ring fragment sterol/hopanoid fragment	
161.13	130	C12H17+	42.07	Sterol/hopanoid fragment	
163.15	65	C12H19+	20.99	Sterol/hopanoid fragment	
175.15	90	C13H19+	12.13	Hopanoid fragment	
177.16	41	C13H21+	-7.48	Hopanoid fragment	
189.17	34	C14H21+	14.27	Hopanoid fragment	
191.18	42	C14H23+	21.60	Hopanoid fragment (norhopane/hopene)	
203.18	31	C15H23+	-3.54	Sterol/hopanoid fragment	
205.19	44	C15H25+	-29.85	Hopanoid fragment	
257.22	101	C19H29+	-18.76	Sterol fragment	
259.25	17	C19H31+	34.04	Sterol fragment	
269.20	29	C19H25O+	22.14	Sterol fragment	
367.33	189	C27H43+	-18.99	Cholestadienol [M + H - H2O]+/cholestadiene [M - H]+/hopene fragment	Cholestadienol/cholestadiene (C27H44O)/(C27H44)
368.34	166	C27H44+	-1.96	Cholestadiene [M]+	
383.33	206	C27H43O+	60.22	Cholestadienol [M - H]+	
369.35	219	C27H45+	4.61	Cholesterol [M + H - H2O]+/cholestene [M - H]+/norhopane & hopene fragment	Cholesterol (C27H46O) Cholestene (C27H46)
385.35	111	C27H45O+	1.85	[M - H]+	
386.37	67	C27H46O+	33.51	[M]+	
381.35	34	C28H45+	0.72	[M + H - H2O]+	Ergostadienol (C28H46O)
397.34	110	C28H45O+	16.47	[M - H]+	
383.33	186	C28H47+	8.37	[M + H -H2O]+ ergostenol/ergostene [M - H]+	Ergostenol (C28H44O) Ergostene (C28H48)
395.33	147	C28H43O+	26.19	[M - H]+	
400.37	21	C28H48O+	-1.79	[M]+	
397.33	22	C29H49+	4.46	[M + H -H2O]+ stignasterol/[M - H]+ stigmastene	Stigmasterol (C29H50O)
414.37	16	C29H50O+	14.56	[M]+	Stigmastene (C29H50)
399.40	11	C29H51+	6.57	[M - H]+	Stigmastane (C29H52)

Table S8. Fragments of N-bearing compounds characterized in sample 95E5/3 using ToF-SIMS.

Main N-bearing ions			
m/z	Intensity (cps)	Ion	error (ppm)
18.03	183	NH_4^+	42.63
26.00	34915	CN^-	101.43
42.00	24837	CNO^-	49.55

Amines and amine adducts			
m/z	Intensity (cps)	M^+	error (ppm)
42.03	1220	C2H4N+	-8.90
44.05	1660	C2H6N+	-0.55
46.07	87	C2H8N+	-32.60
58.07	7056	C3H8N+	72.46

N adducts				
m/z	Intensity (cps)	$[M+N]^+$	M	error (ppm)
296.33	170	C20H42N+	C20H42	-2.62
324.35	31	C22H46N+	C22H46	-40.16
338.38	23	C23H48N+	C23H48	3.91
352.39	30	C24H50N+	C24H50	-6.67
366.40	36	C25H52N+	C25H52	-9.98
380.42	68	C26H54N+	C26H54	-9.60
408.45	30	C28H58N+	C28H58	-9.78
422.47	31	C29H60N+	C29H60	-15.96
464.50	25	C32H66N+	C32H66	-32.31
504.59	91	C35H70N+	C35H70	85.37
562.60	57	C39H80N+	C39H80	-48.33

Trialkylamines formed by NH_4^+ adducts				
m/z	Intensity (cps)	$[M+NH_4]^+$	Compound	error (ppm)
170.20	42	C11H24N+	4-Propyloct-7-en-3-ylazanium	53.62
284.33	126	C19H42N+	Cetrimonium	-19.40
368.42	85	C25H54N+	Docosyltrimethylaminium	-36.63
494.56	136	C34H72N+	Dimethylmyristylstearylammonium	-14.72
522.59	518	C36H76N+	Dimethyl(tetratriacontyl)azanium	-11.21
550.62	679	C38H80N+	trimethyl(pentatriacontyl)azanium	-18.92

Table S9. Organics identified by GC-MS in sample 95E5/2 and by ToF-SIMS in sample 95E5/3 as well as their possible sources.

Sample	Technique	Organics	Taxa
95E5/2	GC-MS	Alkanols, Long-chain (>C22) n-alkanols	Terrestrial higher plants [6, 7]
		Alkanols, Short-chain (<C22) saturated and unsaturated alkanols	Microbes [8, 9]
		FAs, 10Me-C11 and 10Me-C16	Actinomycetes [10, 11]
		FAs, Iso and anteiso methyl-branched saturated FAs	Bacteria [12, 13]
		FAs, Iso-branched C15:0i and C17:0i; or anteiso-branched C15:0a and C17:0a	Gram-positive bacteria [13, 14]
		FAs, C18:1ω9	Fungi or Gram-positive bacteria [15]
		Sterols, Stigmastanol	Vascular plants [16, 17]
		Sterols, Campesterol	Plants [18]
95E5/2 and 95E5/3	GC-MS and ToF-SIMS	FAs, n-C16:0, n-C18:0 and n-C14:0	Bacteria [13]
		FAs, Even-carbon-numbered with long chain lengths (>C20)	Higher plant inputs [19, 20]
		FAs, Saturated C24:0 (Tetracosanoic acid), C26:0 (Hexacosanoic acid), and C28:0 (Octacosanoic acid)	Vascular plant [21]
		Sterols in general	Eukaryotes [22]
		Sterols, Cholesterol	Fungi and algae, rarely found in vascular plants [6, 23]

		Sterols, Stigmasterol	Higher plants [24, 25]
95E5/3	ToF-SIMS	Hopanoids fragments	Aerobic and anaerobic bacteria [26-28]
		Sterols, Ergosterol	Fungi [6, 29]
		Wax esters	Bacteria (e.g. Acinetobacter, Moraxella, Micrococcus, Fundibacter, Neisseria, Marinobacter, Pseudomonas and inomycetes) [30-33]
		Glycerides, DG (30:0) to DG (36:0) together with FAs n-C14 to n-C18	Bacteria and fungi [34]

References

1. van Dongen, B.E.; Semiletov, I.; Weijers, J.W.H.; Gustafsson, Ö., Contrasting lipid biomarker composition of terrestrial organic matter exported from across the Eurasian Arctic by the five great Russian Arctic rivers. *Global Biogeochem. Cycles* **2008,** *22* (1).
2. Herrera-Herrera, A.V.; Leierer, L.; Jambrina-Enríquez, M.; Connolly, R.; Mallol, C., Evaluating different methods for calculating the Carbon Preference Index (CPI): Implications for palaeoecological and archaeological research. *Org. Geochem.* **2020,** *146*, 104056.
3. Rielley, G.; Collier, R.J.; Jones, D.M.; Eglinton, G., The biogeochemistry of Ellesmere Lake, UK—I: source correlation of leaf wax inputs to the sedimentary lipid record. *Org. Geochem.* **1991,** *17* (6), 901-912.
4. Grimalt, J.; Albaigés, J., Sources and occurrence of C12 C22n-alkane distributions with even carbon-number preference in sedimentary environments. *Geochim. Cosmochim. Acta* **1987,** *51* (6), 1379-1384.
5. Sánchez-García, L.; Vonk, J.E.; Charkin, A.N.; Kosmach, D.; Dudarev, O.V.; Semiletov, I.P.; Gustafsson, Ö., Characterisation of Three Regimes of Collapsing Arctic Ice Complex Deposits on the SE Laptev Sea Coast using Biomarkers and Dual Carbon Isotopes. *Permafrost and Periglacial Processes* **2014,** *25* (3), 172-183.
6. Kolattukudy, P.E., Chemistry and biochemistry of natural waxes. **1976**.
7. Sargent, J.; Falk-Petersen, S., Ecological investigations on the zooplankton community in Balsfjorden, Northern Norway: lipids and fatty acids in Meganyctiphanes norvegica, Thysanoessa raschi and T. inermis during mid-winter. *Mar. Biol.* **1981,** *62*, 131-137.

8. Robinson, N.; Cranwell, P.; Finlay, B.; Eglinton, G., Lipids of aquatic organisms as potential contributors to lacustrine sediments. *Org. Geochem.* **1984,** *6*, 143-152.
9. Volkman, J.K.; Barrett, S.M.; Blackburn, S.I., Eustigmatophyte microalgae are potential sources of C29 sterols, C22–C28 n-alcohols and C28–C32 n-alkyl diols in freshwater environments. *Org. Geochem.* **1999,** *30* (5), 307-318.
10. Cantrell, S.A.; Lodge, D.J.; Cruz, C.A.; García, L.M.; Pérez-Jiménez, J.R.; Molina, M., Differential abundance of microbial functional groups along the elevation gradient from the coast to the Luquillo Mountains. *Ecological Bulletins* **2013,** (54), 87-100.
11. Kühn, J.; Schweitzer, K.; Ruess, L., Diversity and specificity of lipid patterns in basal soil food web resources. *PLoS One* **2019,** *14* (8), e0221102.
12. Harwood, J.; Russell, N., *Lipids in Plants and Microbes*. George Allen and Unwin: London, UK, 1984.
13. Kaneda, T., Iso-and anteiso-fatty acids in bacteria: biosynthesis, function, and taxonomic significance. *Microbiol. Mol. Biol. Rev.* **1991,** *55* (2), 288-302.
14. Quideau, S.A.; McIntosh, A.C.; Norris, C.E.; Lloret, E.; Swallow, M.J.; Hannam, K., Extraction and analysis of microbial phospholipid fatty acids in soils. *JoVE* **2016,** (114), e54360.
15. Ruess, L.; Chamberlain, P.M., The fat that matters: soil food web analysis using fatty acids and their carbon stable isotope signature. *Soil Biol. Biochem.* **2010,** *42* (11), 1898-1910.
16. Baker, E.A., Chemistry and morphology of plant epicuticular waxes. *The plant cuticle* **1982**, 139-166.
17. Bianchi, G., Plant waxes. *Waxes: chemistry, molecular biology and functions* **1995,** *6*, 175-222.
18. Du, Y.; Fu, X.; Chu, Y.; Wu, P.; Liu, Y.; Ma, L.; Tian, H.; Zhu, B., Biosynthesis and the roles of plant sterols in development and stress responses. *Int. J. Mol. Sci.* **2022,** *23* (4), 2332.
19. Kolattukudy, P., Cutin, suberin, and waxes. In *Lipids: structure and function*, Elsevier: 1980; pp 571-645.
20. Colombo, J.; Silverberg, N.; Gearing, J., Lipid biogeochemistry in the Laurentian Trough: I—fatty acids, sterols and aliphatic hydrocarbons in rapidly settling particles. *Org. Geochem.* **1996,** *25* (3-4), 211-225.
21. Lebreton, B.; Richard, P.; Galois, R.; Radenac, G.; Pfléger, C.; Guillou, G.; Mornet, F.; Blanchard, G.F., Trophic importance of diatoms in an intertidal Zostera noltii seagrass bed: Evidence from stable isotope and fatty acid analyses. *Estuarine, Coastal Shelf Science* **2011,** *92* (1), 140-153.
22. Peters, K.E.; Walters, C.C.; Moldowan, J.M., *The biomarker guide: Volume 1, Biomarkers and isotopes in the environment and*

human history. Cambridge University Press: Cambridge, UK, 2007.
23. Awad, A.; Majcherczyk, A.; Schall, P.; Schröter, K.; Schöning, I.; Schrumpf, M.; Ehbrecht, M.; Boch, S.; Kahl, T.; Bauhus, J., Ectomycorrhizal and saprotrophic soil fungal biomass are driven by different factors and vary among broadleaf and coniferous temperate forests. *Soil Biol. Biochem.* **2019,** *131*, 9-18.
24. Volkman, J.K., A review of sterol markers for marine and terrigenous organic matter. *Org. Geochem.* **1986,** *9* (2), 83-99.
25. Hartmann, M.-A., Plant sterols and the membrane environment. *Trends Plant Sci.* **1998,** *3* (5), 170-175.
26. Werne, J.P.; Baas, M.; Sinninghe Damsté, J.S., Molecular isotopic tracing of carbon flow and trophic relationships in a methane‐supported benthic microbial community. *Limnol. Oceanogr.* **2002,** *47* (6), 1694-1701.
27. Stadnitskaia, A.; Muyzer, G.; Abbas, B.; Coolen, M.J.L.; Hopmans, E.C.; Baas, M.; Van Weering, T.C.E.; Ivanov, M.K.; Poludetkina, E.; Damsté, J.S.S., Biomarker and 16S rDNA evidence for anaerobic oxidation of methane and related carbonate precipitation in deep-sea mud volcanoes of the Sorokin Trough, Black Sea. *Mar. Geol.* **2005,** *217* (1-2), 67-96.
28. Blumenberg, M.; Krüger, M.; Nauhaus, K.; Talbot, H.M.; Oppermann, B.I.; Seifert, R.; Pape, T.; Michaelis, W., Biosynthesis of hopanoids by sulfate‐reducing bacteria (genus Desulfovibrio). *Environ. Microbiol.* **2006,** *8* (7), 1220-1227.
29. Ruzicka, S.; Edgerton, D.; Norman, M.; Hill, T., The utility of ergosterol as a bioindicator of fungi in temperate soils. *Soil Biol. Biochem.* **2000,** *32* (7), 989-1005.
30. Fixter, L.M.; Nagi, M.N.; Mccormack, J.G.; Fewson, C.A., Structure, distribution and function of wax esters in Acinetobacter calcoaceticus. *Microbiology* **1986,** *132* (11), 3147-3157.
31. Wältermann, M.; Hinz, A.; Robenek, H.; Troyer, D.; Reichelt, R.; Malkus, U.; Galla, H.J.; Kalscheuer, R.; Stöveken, T.; Von Landenberg, P., Mechanism of lipid‐body formation in prokaryotes: how bacteria fatten up. *Mol. Microbiol.* **2005,** *55* (3), 750-763.
32. Ishige, T.; Tani, A.; Sakai, Y.; Kato, N., Wax ester production by bacteria. *Curr. Opin. Microbiol.* **2003,** *6* (3), 244-250.
33. Wältermann, M.; Steinbüchel, A., Neutral lipid bodies in prokaryotes: recent insights into structure, formation, and relationship to eukaryotic lipid depots. *J. Bacteriol.* **2005,** *187* (11), 3607-3619.
34. Fernández-Remolar, D.C.; Carrizo, D.; Harir, M.; Huang, T.; Amils, R.; Schmitt-Kopplin, P.; Sánchez-García, L.; Gomez-Ortiz, D.; Malmberg, P., Unveiling microbial preservation under hyperacidic and oxidizing conditions in the Oligocene Rio Tinto deposit. *Sci. Rep.* **2021,** *11* (1), 21543.
